# Supplementary material for: Predicting mutational routes to new adaptive phenotypes
Source: eLife. 2019 Jan 8;8:e38822. doi: 10.7554/eLife.38822 (PMC6324874; doi:10.7554/eLife.38822)
Supplement: Supplementary file 1. [file elife-38822-supp1.pdf]

Differential equations for WSP, AWS, & MWS pathways

WSP

$$\frac{d[ABD]}{dt} = r_2F^*[ABDm] - r_1[ABD][Cm]$$
$$\frac{d[ABDm]}{dt} = r_1[ABD][Cm] - r_2F^*[ABDm] - r_3S[ABDm] + r_4E[ABDm^*]$$
$$\frac{d[ABDm^*]}{dt} = -r_4E[ABDm^*] + r_3S[ABDm]$$
$$\frac{dE^*}{dt} = r_4E[ABDm^*] - r_5E^*R - r_6E^*F$$
$$\frac{dR^*}{dt} = r_5RE^*$$
$$\frac{dE}{dt} = r_6E^*F - r_4E[ABDm^*] + r_5RE^*$$
$$\frac{dF}{dt} = -r_6E^*F + r_2F^*[ABDm]$$
$$\frac{dF^*}{dt} = r_6E^*F - r_2F^*[ABDm]$$
$$\frac{dR}{dt} = -r_5RE^* - .01R$$

AWS

$$\frac{dX}{dt} = -r_2XO^* - r_3XR$$
$$\frac{d[XR]}{dt} = r_3XR$$
$$\frac{dO}{dt} = -r_1SO$$
$$\frac{dO^*}{dt} = r_1SO - r_2XO^*$$
$$\frac{d[OX]}{dt} = r_2O^*X$$
$$\frac{dR}{dt} = -r_3XR - r4RR$$
$$\frac{d[RR]}{dt} = r_4RR$$

MWS

$$\begin{aligned}\frac{dG}{dt} &= -r_1 GS\\ \frac{dG^*}{dt} &= r_1 GS - r_2 G^* E - r_3 G^* C\\ \frac{dE}{dt} &= -r_2 G^* E\\ \frac{d[GE]}{dt} &= r_2 G^* E\\ \frac{dC}{dt} &= -r_3 G^* C\\ \frac{d[GC]}{dt} &= r3 G^* C\end{aligned}$$

Julia code for WSP, AWS, and MWS differential equations

The following three functions implement the differential equation model (ODE model) for the WSP, AWS, and MWS pathways.

```
using ODE,StatsBase,MAT,HDF5,JLD

# code for WSP differential equations
function lindodeWSP(t,y)
   # convert y to reactants for ease of reading
   ABD=y[1];
   ABDm=y[2];
   ABDmp=y[3];
   Ep=y[4];
   Rp=y[5];
   E=y[6];
   F=y[7];
   Fp=y[8];
   R=y[9];
   # pull reaction rates from rs variable
   r1=rs[1];r2=rs[2];r3=rs[3];r4=rs[4];r5=rs[5];r6=rs[6];
   # compute derivatives, i.e. y'
   yp=zeros(size(y));
   yp[1]=r2*Fp*ABDm-r1*ABD*Cm; # dABD/dt
   yp[2]=r1*ABD*Cm-r2*Fp*ABDm-r3*S*ABDm+r4*E*ABDmp; # dABDm/dt
   yp[3]=-r4*E*ABDmp + r3*S*ABDm ; # dABDmp/dt
   yp[4]=r4*E*ABDmp - r5*Ep*R -r6*Ep*F ; # dEp/dt
   yp[5]=r5*R*Ep ; # dRp/dt
   yp[6]=r6*Ep*F-r4*E*ABDmp+r5*R*Ep ; # dE/dt
   yp[7]=-r6*Ep*F +r2*Fp*ABDm ; # dF/dt
   yp[8]=r6*Ep*F -r2*Fp*ABDm ; # dFp/dt
   yp[9]=-r5*R*Ep-.01*R; # dR/dt
   return yp
end

# code for AWS differential equations
```

```
function lindodeAWS(t,y)
   # convert y to reactants for ease of reading
   X=y[1];
   XR=y[2];
   O=y[3];
   Op=y[4];
   OX=y[5];
   R=y[6];
   RR=y[7];
   # pull reaction rates from rs variable
   r1=rs[1];r2=rs[2];r3=rs[3];r4=rs[4];
   # compute derivatives, i.e. y'
   yp=zeros(size(y));
   yp[1]=-r2*X*Op-r3*X*R; # dX/dt
   yp[2]=r3*X*R; # dXR/dt
   yp[3]=-r1*S*O; # dO/dt
   yp[4]=r1*S*O-r2*X*Op; # dOp/dt
   yp[5]=r2*Op*X; # dOX/dt
   yp[6]=-r3*X*R-r4*R*R; # dR/dt
   yp[7]=r4*R*R; # dRR/dt
   return yp;
end

# code for MWS differential equations
function lindodeMWS(t,y)
   # convert y to reactants for ease of reading
   G=y[1];
   Gp=y[2];
   E=y[3];
   GE=y[4];
   C=y[5];
   GC=y[6];
   # pull reaction rates from rs variable
   r1=rs[1];r2=rs[2];r3=rs[3];
   # compute derivatives, i.e. y'
   yp=zeros(size(y));
   yp[1]=-r1*G*S; # dG/dt
   yp[2]=r1*G*S-r2*Gp*E-r3*Gp*C;# dGp/dt
   yp[3]= -r2*Gp*E;# dE/dt
   yp[4]= r2*Gp*E;# dGE/dt
   yp[5]=-r3*Gp*C;# dC/dt
   yp[6]=r3*Gp*C; # dGC/dt
   return yp;
end
```

Julia code for running differential equation solvers

We include the code for implementing our Bayesian sampling method. The colored sections correspond to statements that make it specific to WSP (blue), AWS (red), or MWS (green). It was run in julia version 0.4.3.

```
# Variables for reactions
numrxns=6; # number of reaction rates for WSP
```

```
numrxns=4; # number of reaction rates for AWS
numrxns=3; # number of reaction rates for MWS
rs=rand(numrxns); # establish variable scope, will be reaction rates later
rs_save=copy(rs); # establish variable scope, will be a saved version of reaction rates later
totnumruns=3.^length(rs); # all possible combinations for reaction rates (down,nothing, up)
v=zeros(length(rs)); # establish variable scope (used to alter reaction rates)
S=0;Cm=0; # initialize constants used in differential equations
indexWS=5; # reactant corresponding to WS in WSP
indexWS=7; # reactant corresponding to WS in AWS
indexWS=6; # reactant corresponding to WS in MWS

# Variables for running ODE solver
testnums=1000; # number of runs
yorig=zeros(testnums); # storage for baseline WS production
yout=0; # establish variable scope
tf=1.0; # establish variable scope
tftimes=1.0; # establish variable scope

numreactants=9; # number of reactants
numreactants=7; # number of reactants
numreactants=6; # number of reactants
init=10*rand(numreactants); # establish variable scope


# Variables for storing data
res=-1*ones(totnumruns,testnums); # storage for altered WS production
numfinished=1; # counter for runs completed

# Code for Bayesian sampling method
while numfinished<=testnums
   done=0;
   try
      println(numfinished) # keeps track of how many sims have been done
      # Sample concentrations and rates to establish a baseline amount of WS production
      rs=10.^(4*rand(numrxns)-2); # sample reaction rates from [.01,100]
      rs_save=copy(rs); # saved copy as a reference when altering later
      init=10*rand(numreactants); # sample initial concentrations for reactants from [0,10]
      S=10*rand(); # sample initial concentration for constant reactant of signal (S) from [0,10]
      Cm=10*rand(); # sample initial concentration for constant reactant Cm from [0,10]

      # ODE solver for baseline
      tf=1.0; # initial time for ode solver
      dst=100; # initial distance, used to determine solution converged
      tol=10^(-8.0); # tolerance for ODE solver
      while dst>tol
         tout, yout = ode45(lindodeWSP, init, [0.0 ,tf]);
         tout, yout = ode45(lindodeAWS, init, [0.0 ,tf]);
         tout, yout = ode45(lindodeMWS, init, [0.0 ,tf]);
         dst=sum((yout[end-1]-yout[end]).^2); # Euclidean distance in final step of solution
         yorig[numfinished]=yout[end][indexWS]; # reactant corresponding to WS
         tftimes=tout[end]; # final time
         tf*=2;
      end
      done=1; # successful completion of try loop
   end
   if done==1 # baseline WS production is established, now sample changes/mutations
      i0=1; # counter for completed changes
      cct=0; # counter for total number of attempts
```

```
      while i0<=totnumruns;
         cct+=1;
         println([numfinished cct]) # report status for tracking progress
         # Alter reaction rates
         num=i0-1; # used for determining which rates change down/none/up
            for i1=length(rs)-1:-1:0;
                  v[i1+1]=floor(num/(3^i1)); # v is num into base 3 number
                  num=num-v[i1+1]*3^i1;
            end
         v+=1;
         facs=[10.^(-2*rand()), 1, 10.^(2*rand())]; # factors to alter rxn rates [.01,1] down, 1 none, [1,100] up
         for i1=1:length(rs)
            rs[i1]=facs[v[i1]]*rs_save[i1]; # alter reaction rates
         end
         try
            tout, yout = ode45(lindodeWSP, init, [0.0,tftimes]);
            tout, yout = ode45(lindodeAWS, init, [0.0,tftimes]);
            tout, yout = ode45(lindodeMWS, init, [0.0,tftimes]);
            if abs(tout[end]-tftimes)<.01 # ODE solver finished
                     res[i0,numfinished]=yout[end][indexWS]; # store amount of WS produced
               i0+=1
            end
         end
         if cct>10000
            i0=2*totnumruns; # baseline and sampling occurred in space with poorly conditioned ODEs, try again
         end
      end
      if i0<2*totnumruns # successful
         numfinished+=1;
         # Save data to a file for checking in MATLAB
         file=matopen("pathway_results_temp.mat","w")
         write(file,"res",res); altered WS production
         write(file,"yorig",yorig); baseline WS production
         close(file);
      end
      end
end

# Save data to a file for processing in MATLAB
file=matopen("pathway_results_complete_WSP.mat","w")
file=matopen("pathway_results_complete_AWS.mat","w")
file=matopen("pathway_results_complete_MWS.mat","w")
write(file,"res",res); altered WS production
write(file,"yorig",yorig); baseline WS production
close(file);
```

MATLAB code for interpreting saved data WSP vs MWS

This code shows how the data from the julia code is analyzed and transformed into the contour plots shown in the paper.

```
% create record of how parameters change down, none, up for WSP
rs1=rand(1,6);
```

```
totnumruns=3.^length(rs1);
paramsWSP=zeros(totnumruns,length(rs1));
v=zeros(size(rs1));
i0=1;
while i0<=totnumruns;
    num=i0-1;
    for i1=length(rs1)-1:-1:0;
            v(i1+1)=floor(num/(3^i1));
            num=num-v(i1+1)*3^i1;
    end
    v=v+1;
    paramsWSP(i0,:)=v;
    i0=i0+1;
end
% create record of how parameters change down, none, up for MWS
rs1=rand(1,3);
totnumruns=3.^length(rs1);
paramsMWS=zeros(totnumruns,length(rs1));
v=zeros(size(rs1));
i0=1;
while i0<=totnumruns;
    num=i0-1;
    for i1=length(rs1)-1:-1:0;
            v(i1+1)=floor(num/(3^i1));
            num=num-v(i1+1)*3^i1;
    end
    v=v+1;
    paramsMWS(i0,:)=v;
    i0=i0+1;
end

% Load data
load pathway_results_complete_MWS.mat
resMWS=res;
yorigMWS=yorig';
clear res yorig
load pathway_results_complete_WSP.mat
resWSP=res;
yorigWSP=yorig';

% Variables and data storage to compare likelihood of pathways
numsampWSP=size(resWSP,2); % in case want to use fewer samples
numsampMWS=size(resMWS,2); % in case want to use fewer samples

perange=10.^[-7:.5:-1]; % range for probability enabling mutations
pdrange=10.^[-7:.5:-1]; % range for probability disabling mutations
pemat=zeros(length(perange),length(pdrange)); % matrix for plotting data and reference
pdmat=zeros(size(pemat)); % matrix for plotting data and reference
psummatWSP=zeros(size(pemat)); % matrix for probability WSP used
psummatMWS=zeros(size(pemat)); % matrix for probability MWS used
tol=0;
% Code to compare likelihood of pathways
for i0=1:length(perange)
    for j0=1:length(pdrange)
        % Retrieve probabilities
        pe=perange(i0);
        pd=pdrange(j0);
        pemat(i0,j0)=pe;
```

```
        pdmat(i0,j0)=pd;
        % WSP computation
        pmatforr=ones(6,1)*[pd 1-pe-pd pe];
        psum=0; % total sum of (prob of rxn changes) X (number of times WS produced)
        for i1=1:size(resWSP,1)
            probevent=1; % initialize, probability to get combination of down, none, up for rxns
            for j1=1:6;
                probevent=probevent*pmatforr(j1,paramsWSP(i1,j1)); % multiply by prob of each change
                    end
            psum=psum+probevent*sum(resWSP(i1,1:numsampWSP)>yorigWSP(1:numsampWSP)+tol)/numsampWSP;
        end
        psummatWSP(i0,j0)=psum;

        % MWS computation
        pmatforr=ones(3,1)*[pd 1-pe-pd pe];
        psum=0; % total sum of (prob of rxn changes) X (number of times WS produced)
        for i1=1:size(resMWS,1)
            probevent=1; % initialize, probability to get combination of down, none, up for rxns
            for j1=1:3;
                probevent=probevent*pmatforr(j1,paramsMWS(i1,j1)); % multiply by prob of each change
            end
            psum=psum+probevent*sum(resMWS(i1,1:numsampMWS)>yorigMWS(1:numsampMWS)+tol)/numsampMWS;
        end
        psummatMWS(i0,j0)=psum;
    end
end

% Plot data
close all
figure
contourf(pemat,pdmat,log2(psummatWSP./psummatMWS),400,'LineStyle','None')
set(gca,'xScale','log','yScale','log','TickLength',[.025 .025],'LineWidth',3);
set(gca,'FontSize',18,'xTick',10.^[-7:1:-1],'yTick',10.^[-7:1:-1]);
xlabel('Probability of enabling change','FontSize',24);
ylabel('Probability of disabling change','FontSize',24);
c=colorbar('FontSize',18);
c.Label.String='log_2 ratio probability WSP/MWS';
colormap jet;
axis square
eval(['print -f1 -depsc -r300 WSP_vs_MWS_contour.eps']);
```

MATLAB code for interpreting saved data WSP vs AWS

```
% create record of how parameters change down, none, up for WSP
rs1=rand(1,6);
totnumruns=3.^length(rs1);
paramsWSP=zeros(totnumruns,length(rs1));
v=zeros(size(rs1));
i0=1;
while i0<=totnumruns;
    num=i0-1;
    for i1=length(rs1)-1:-1:0;
            v(i1+1)=floor(num/(3^i1));
```

```
            num=num-v(i1+1)*3^i1;
    end
    v=v+1;
    paramsWSP(i0,:)=v;
    i0=i0+1;
end
% create record of how parameters change down, none, up for AWS
rs1=rand(1,4);
totnumruns=3.^length(rs1);
paramsAWS=zeros(totnumruns,length(rs1));
v=zeros(size(rs1));
i0=1;
while i0<=totnumruns;
    num=i0-1;
    for i1=length(rs1)-1:-1:0;
            v(i1+1)=floor(num/(3^i1));
            num=num-v(i1+1)*3^i1;
    end
    v=v+1;
    paramsAWS(i0,:)=v;
    i0=i0+1;
end

% Load data
load pathway_results_complete_AWS.mat
resAWS=res;
yorigAWS=yorig';
clear res yorig
load pathway_results_complete_WSP.mat
resWSP=res;
yorigWSP=yorig';

% Variables and data storage to compare likelihood of pathways
numsampWSP=size(resWSP,2); % in case want to use fewer samples
numsampAWS=size(resAWS,2); % in case want to use fewer samples

perange=10.^[-7:.5:-1]; % range for probability enabling mutations
pdrange=10.^[-7:.5:-1]; % range for probability disabling mutations
pemat=zeros(length(perange),length(pdrange)); % matrix for plotting data and reference
pdmat=zeros(size(pemat)); % matrix for plotting data and reference
psummatWSP=zeros(size(pemat)); % matrix for probability WSP used
psummatAWS=zeros(size(pemat)); % matrix for probability MWS used
tol=0;
fac=5; % factor increase of mutation because of hotspot

% Code to compare likelihood of pathways
for i0=1:length(perange)
    for j0=1:length(pdrange)
        % Retrieve probabilities
        pe=perange(i0);
        pd=pdrange(j0);
        pemat(i0,j0)=pe;
        pdmat(i0,j0)=pd;
        % WSP computation
        pmatforr=ones(6,1)*[pd 1-pe-pd pe];
        psum=0; % total sum of (prob of rxn changes) X (number of times WS produced)
        for i1=1:size(resWSP,1)
            probevent=1; % initialize, probability to get combination of down, none, up for rxns
```

```
            for j1=1:6;
                probevent=probevent*pmatforr(j1,paramsWSP(i1,j1)); % multiply by prob of each change
                    end
            psum=psum+probevent*sum(resWSP(i1,1:numsampWSP)>yorigWSP(1:numsampWSP)+tol)/numsampWSP;
        end
        psummatWSP(i0,j0)=psum;

        % AWS computation
        pmatforr=ones(4,1)*[pd 1-pe-pd pe];
        pmatforr(4,:)=[.5*pd 1-.5*pd-.5*pe .5*pe]; % because only reactant in dimerization
        pmatforr(3,:)=[fac*pd 1-fac*pd-fac*pe fac*pe]; % effect of hotspot
        pmatforr(2,:)=[fac*pd 1-fac*pd-fac*pe fac*pe]; % effect of hotspot
        psum=0; % total sum of (prob of rxn changes) X (number of times WS produced)
        for i1=1:size(resAWS,1)
            probevent=1; % initialize, probability to get combination of down, none, up for rxns
            for j1=1:4;
                probevent=probevent*pmatforr(j1,paramsAWS(i1,j1)); % multiply by prob of each change
            end
            psum=psum+probevent*sum(resAWS(i1,1:numsampAWS)>yorigAWS(1:numsampAWS)+tol)/numsampAWS;
        end
        psummatAWS(i0,j0)=psum;
    end
end

% Plot data
close all
figure
contourf(pemat,pdmat,log2(psummatWSP./psummatAWS),400,'LineStyle','None')
set(gca,'xScale','log','yScale','log','TickLength',[.025 .025],'LineWidth',3);
set(gca,'FontSize',18,'xTick',10.^[-7:1:-1],'yTick',10.^[-7:1:-1]);
xlabel('Probability of enabling change','FontSize',24);
ylabel('Probability of disabling change','FontSize',24);
c=colorbar('FontSize',18);
c.Label.String='log_2 ratio probability WSP/AWS';
colormap jet;
axis square
eval(['print -f1 -depsc -r300 WSP_vs_AWS_contour.eps']);
```
